# Advances in immunotherapy and targeted therapy for advanced clear-cell renal cell carcinoma: current strategies and future directions

**DOI:** 10.3389/fimmu.2025.1582887

**Published:** 2025-07-03

**Authors:** Bo Wang, Yuchu Xiang, Xudong Liu, Xiaoting Pan, Lang Peng, Jiajia Ma

**Affiliations:** ^1^ Institute of Medical Microbiology and Hygiene, Faculty of Medicine, University of Freiburg, Freiburg, Germany; ^2^ Department of Dermatology, West China Hospital, Sichuan University, Chengdu, China; ^3^ Laboratory of Dermatology, Clinical Institute of Inflammation and Immunology, Frontiers Science Center for Disease-related Molecular Network, West China Hospital, Sichuan University, Chengdu, China; ^4^ Department of Clinical Medicine, Shanghai Medical College, Fudan University, Shanghai, China

**Keywords:** renal cell carcinoma, immunotherapy, targeted therapy, current strategies, future directions

## Abstract

Renal cell carcinoma (RCC), particularly the clear-cell subtype (ccRCC), accounts for 75-85% of kidney cancers and exhibits distinct genetic and biological heterogeneity. While surgical resection remains the mainstay of treatment for localized ccRCC, the persistence of recurrence rates underscores the significant unmet need for effective adjuvant therapies. Recent advancements in immunotherapy and targeted therapies have revolutionized the management of RCC. Immune checkpoint inhibitors have significantly enhanced antitumor immune responses, whereas tyrosine kinase inhibitors (TKIs) and mammalian target of rapamycin (mTOR) inhibitors effectively disrupt angiogenesis and proliferation signaling pathways, respectively. However, non-clear cell RCC subtypes remain understudied due to their rarity and exclusion from major clinical trials. Consequently, this review primarily focuses on ccRCC, aiming to provide a comprehensive and up-to-date overview of the latest advancements in immunotherapy and targeted therapies. By synthesizing current evidence, this review seeks to elucidate the mechanisms of action, clinical efficacy, and limitations of these treatments, while also identifying gaps in knowledge and future research directions. Ultimately, the goal is to offer valuable insights for clinicians and researchers, facilitating the development of optimized, personalized treatment approaches to improve outcomes for ccRCC patients.

## 1 Introduction

Renal cancer remains a common cancer globally, with an estimated 431,288 new cases in 2020 worldwide (1). Recent studies suggest that new favorable subsets of cancers of unknown primary (CUP), including renal cell carcinoma (RCC) CUP, have emerged. This newly recognized clinical entity is treated similarly to RCC and contributes to the currently increasing incidence of RCC (2).The preponderance of kidney cancers comprises RCC, predominantly clear-cell RCC (ccRCC) occupying 75-85% of instances, trailed by papillary, chromophobe, medullary, and collecting duct subtypes. These subgroups of RCCs differ from each other in genetics, biology, and behavior (3). Localized ccRCC is conventionally managed through surgical excision, encompassing both radical and partial nephrectomy approaches, aimed at achieving definitive cure (4). However, the recurrence rate of RCC at 5-years is significant, varying from 2.2% to 58.1% depending on risk factors such as tumor size, histology, and other clinical features (5). Therefore, the necessity for adjuvant and/or neoadjuvant therapies to mitigate recurrence risks and enhance survival outcomes underscores a crucial, as yet unfulfilled, clinical requirement. Apart from surgical interventions, a diverse array of therapeutic modalities has emerged recently to arrest tumor progression and hinder RCC metastases, embracing radiotherapy, chemotherapy, immunological therapies, as well as precision-guided targeted therapies, among others (6).

The profound comprehension of the immune system and cancer’s molecular underpinnings has spawned immunotherapy and targeted therapy, respectively, revolutionizing contemporary therapeutic strategies for renal cancer (7). These innovative approaches offer more treatment options and better outcomes for kidney cancer patients.

Immunotherapy has significantly progressed, utilizing immune checkpoint inhibitors such as Programmed Cell Death Protein-1/Programmed Death-Ligand 1(PD-1/PD-L1) and Cytotoxic T-lymphocyte-associated protein 4(CTLA-4) antagonists, among others, to amplify the immune system’s capacity to counteract tumor growth (8). In addition, targeted therapies further complement this arsenal. For example, Sunitinib, a tyrosine kinase inhibitor (TKI), impedes tumor angiogenesis and expansion through Vascular Endothelial Growth Factor Receptor(VEGFR) disruption (9), while Everolimus and Temsirolimus, mammalian target of rapamycin (mTOR) inhibitors, hinder tumor growth and proliferation by modulating the mTOR signaling cascade (10–12). It should be noted that TKI monotherapy remains a suitable first-line option for patients ineligible for immunotherapy, as supported by the STAR trial. It also offers benefits as a second-line treatment. Although treatment breaks may be considered, caution is warranted, as these patients typically experience shorter progression-free survival compared to those receiving first-line TKI therapy (13).

Given the heterogeneous histology and the rarity of each individual subtype within non-clear cell RCCs (nccRCCs), these tumors have traditionally been under represented in clinical trials, resulting in a lack of well-studied treatment options specifically tailored to them (14). Consequently, this review primarily focuses on ccRCC, provides a comprehensive overview of the immunotherapy and targeted therapies for this specific subtype of kidney cancer.

## 2 Immunotherapy in RCC

### 2.1 Immunotherapy origins

**Figure 1:**
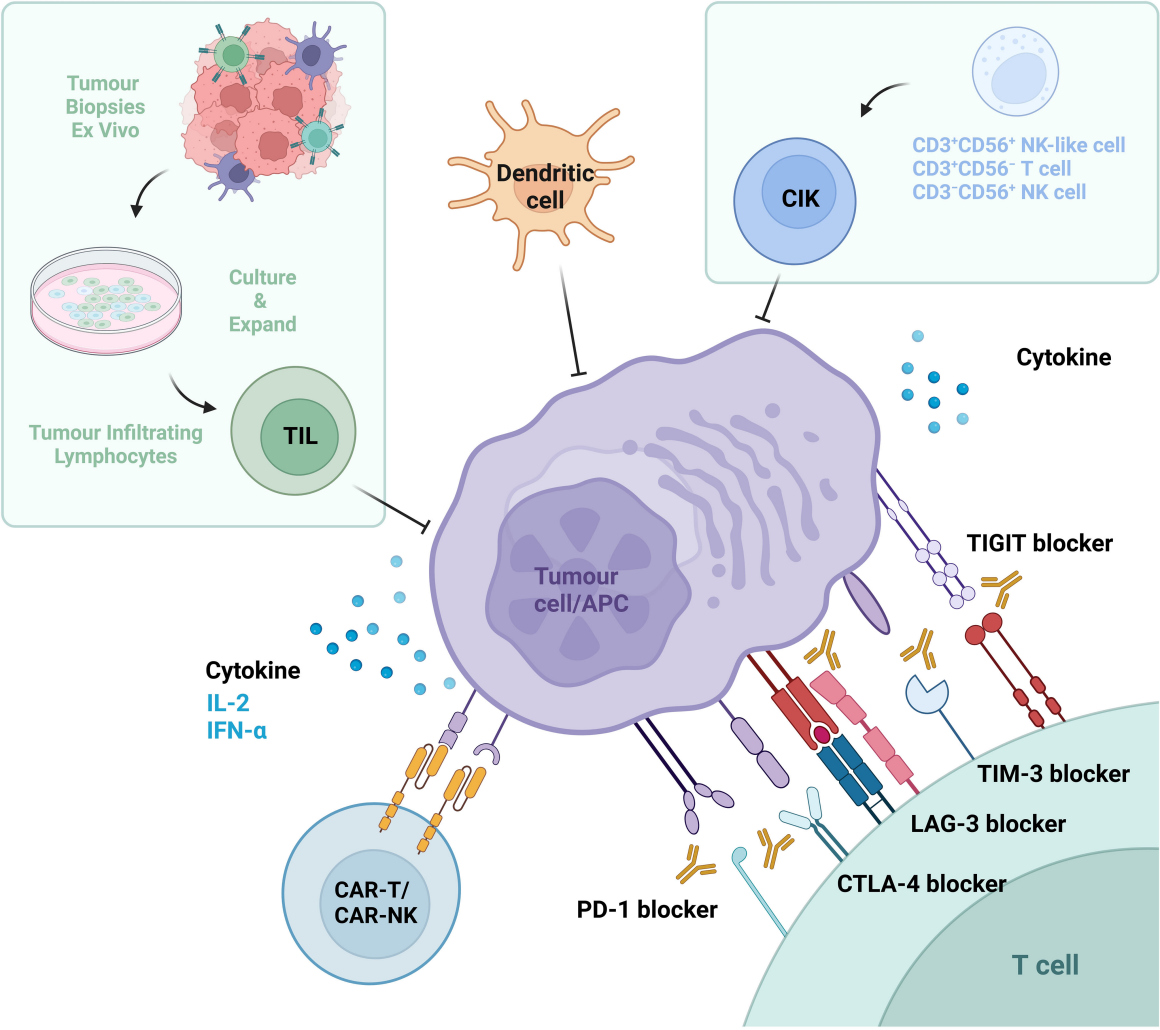
Immunotherapeutic strategies in renal cell carcinoma (RCC). Current immunotherapeutic approaches for RCC encompass three primary categories: (1) Cytokine therapy, involving interleukin-2 (IL-2) and interferon-alpha (IFN-α); (2) Immune checkpoint inhibitors (ICIs), including antibodies targeting coinhibitory receptors such as programmed cell death protein 1 (PD-1), programmed death-ligand 1 (PD-L1), cytotoxic T-lymphocyte-associated protein 4 (CTLA-4), lymphocyte-activation gene 3 (LAG-3), T-cell immunoglobulin and mucin domain-containing protein 3 (TIM-3), and T cell immunoreceptor with Ig and ITIM domains (TIGIT); (3) Adoptive cell therapies, including ex vivo-expanded cytokine-induced killer (CIK) cells, tumor-infiltrating lymphocytes (TILs), genetically engineered chimeric antigen receptor T-cells (CAR-Ts) and natural killer cells (CAR-NKs), as well as dendritic cells (DCs). These modalities collectively aim to enhance intrinsic antitumor immune responses through distinct mechanistic frameworks.

The origins of immunotherapy date back to the late 19th century, when William Coley discovered that bacterial infections (Coley’s toxins) helped treat cancer. The patient’s immune system is provoked by these toxins, facilitating its ability to assault and eradicate cancerous tumor cells (15, 16). The development of modern immunotherapy is based on in-depth research into T cell function and immune checkpoint molecule (17). And immunotherapy works by activating the patient’s own immune system to attack tumor cells, primarily involving cytokine therapy and immune checkpoint inhibitors(ICIs) (**Figure 1**). In addition, with the continuous deepening of research, adoptive cell therapies-including non-gene-modified and gene-modified adoptive cell therapies- along with dendritic cells(DCs) and vaccines have gradually come into the spotlight.

#### 2.1.1 Cytokines

Cytokine therapy, utilizing molecules like interleukin-2 (IL-2) and interferon-alpha (IFN-α), have surfaced as a validated therapeutic strategy for managing metastatic RCC (18–20). Historically, before the advent of targeted therapies, advanced RCC treatment heavily relied on cytokine immunotherapy using either interferon or IL-2 (21). Interferon, an early adopted protein, was administered for RCC treatment, potentiating the immune system’s efficacy in combating neoplastic diseases. Although some patients showed some tumor shrinkage after interferon treatment, the overall response rate was low (approximately 15-20%) and treatment was associated with severe side effects such as flu-like symptoms, fatigue, and depression (22). IL-2, a type of cytokine, stimulates T-lymphocytes and natural killer (NK) cells, enhancing their capacity to eliminate tumor cells. In RCC, select patients have responded favorably to high-dose IL-2 therapy, with some experiencing complete tumor regression (23). However, IL-2 administration is plagued by significant toxicity, particularly severe vascular leak syndrome, thus impeding its broad clinical application (24).

#### 2.1.2 Immune checkpoint inhibitors

Immune checkpoint inhibitors (ICIs), a class of immunotherapy drugs, block checkpoint protein signaling pathways to enhance anti-tumor immune responses by relieving immune suppression (25). Their primary function is to block inhibitory signals, thereby enabling immune cells to mount a more effective tumor-specific cytotoxic response and eliminate cancer cells. Recent advancements highlight the pivotal role of this therapy in the management of ccRCC, particularly in advanced stages (**Table 1**).

**Table 1:** Selected pivotal ICIs involved in RCC management.

Target protein	ICIs	Clinical trial	Phase	Response rate	Status
PD-1	Nivolumab	NCT01668784	3	ORR 25.9%, PFS 4.21 months	Completed
PD-1	Pembrolizumab	NCT03142334	3	OS 72 months	Active, not recruiting
LAG-3 and PD-1	Nivolumab+ Relatlimab	NCT02996110	2	ORR 30%, mDOR 32.57 weeks	Completed
PD-1 and CTLA-4	Nivolumab + Ipilimumab	NCT02231749	3	ORR 41.6%, PFS 11.56	Active, not recruiting
TIM-3	Sabatolimab	NCT02608268	1/2	PFS 1.8 months, OS 4.1 months	Terminated

ORR, Objective response rate; PFS, Progression-free survival; OS, Overall survival.

##### 2.1.2.1 PD-1 inhibitors

In 1992, T. Honjo identified PD-1, also designated as Cluster of Differentiation(CD)279. This transmembrane protein is encoded by the Programmed Cell Death 1 (*PDCD1*) gene (26). T. Honjo and J. Allison made groundbreaking discoveries regarding the role of PD-1 and CTLA-4 inhibition in cancer therapy, which earned them the 2018 Nobel Prize in Physiology or Medicine (27). PD-1 is prominently expressed on tumor-directed T-cells (CD8^+^ cytotoxic T-cells and CD4^+^ T helper1 cells), NK cells, B-cells, monocytes, and DCs. Upon engagement with its ligands, PD-L1 or Programmed Death-Ligand 2(PD-L2), PD-1 activates tyrosine phosphatases (such as Src Homology 2 Domain-Containing Phosphatase-1/2), which dephosphorylate key signaling molecules downstream of the T-cell receptor (TCR), thus inhibiting TCR signaling pathways crucial for T-cell activation. This results in attenuated cell survival signals, impaired cytokine release, and contributes to T-cell exhaustion, reducing immune cell responsiveness and promoting immune evasion. Notably, PD-1 ligands are expressed on both immune cells and tumor cells, allowing tumors to suppress immune responses and evade immune detection (28–30).

PD-1 inhibitors, a class of monoclonal antibodies, serve as immune checkpoint modulators by disrupting inhibitory signals mediated by PD-1 transmembrane proteins on effector immune cells. Currently, Pembrolizumab (Keytruda) and Nivolumab (Opdivo), two anti-PD-1 agents, are utilized in the treatment of ccRCC. Pembrolizumab, the first humanized IgG4 (S228P) monoclonal antibody, received Food and Drug Administration(FDA) approval in 2021 for various cancer indications, including RCC. Nivolumab, another human IgG4 antibody, preceded Pembrolizumab in 2015 as the first FDA-approved checkpoint inhibitor for advanced RCC. Both drugs can elicit adverse effects, such as gastrointestinal distress, muscular discomfort, and fatigue, likely by perturbing physiological immune processes (31–33). Notably, in ccRCC patients, anti-PD-1 therapies exhibit constrained efficacy against central nervous system metastases due to limited blood-brain barrier permeation (34). To mitigate these limitations, research has intensified on combinatorial immunotherapy strategies. In 2018, the FDA endorsed a PD-1/CTLA-4 (Ipilimumab) combination to enhance ccRCC treatment outcomes. Additionally, the potential synergy between Pembrolizumab and the anti-VEGF agent Pazopanib is still under investigation (25).

##### 2.1.2.2 CTLA-4 inhibitors

CTLA-4 (CD152), a pivotal immune checkpoint, modulates antitumor immunity by inhibiting immune responses to cancer. Encoded by the CTLA-4 gene, it acts as a key regulator of immune tolerance. Its role was first discerned in 1987 and further elucidated in the 1990s (28). CTLA-4 is composed of 223 amino acids (35) and is upregulated in tumor-targeting T cells. Upon binding to CD80 (B7-1) or CD86 (B7-2) on antigen-presenting cells or tumor cells, it functions as an inhibitory switch. This interaction triggers the phosphorylation of its cytoplasmic YVKM motif, which recruits Src Homology 2 Domain-Containing Phosphatase-2 and activates Serine/Threonine Protein Phosphatase 2A. This cascade suppresses TCR/CD28-mediated activation of the Phosphatidylinositol 3-kinase (PI3K)/Protein Kinase B (Akt) signaling pathway, thereby limiting TCR signaling that is essential for T-cell activation and cytokine production (27, 36).

Ipilimumab (Yervoy) is an FDA-approved human Immunoglobulin G1 kappa (IgG1κ) monoclonal antibody that specifically targets CTLA-4. While CTLA-4 inhibitors can elicit adverse reactions such as exhaustion and gastrointestinal disturbances, blocking CTLA-4 in melanoma therapy has been associated with severe Immune-Related Adverse Events(AE), including autoimmune complications in visceral organs such as the colon, liver, and endocrine glands (28). To enhance efficacy in resistant patients, the FDA approved the combination of Ipilimumab with Nivolumab as a first-line therapeutic regimen for advanced RCC in adults (37). This synergy arises from their complementary mechanisms of action: Anti-PD-1 therapy revitalizes exhausted effector T cells, thereby enhancing immune responses, while anti-CTLA-4 promotes antigen-specific T-cell activation and priming (38).

##### 2.1.2.3 Lymphocyte-Activation Gene 3 inhibitors

Lymphocyte-Activation Gene 3 (LAG-3), also known as CD223, was first identified in 1990 by Triebel’s team. This 498-amino acid transmembrane protein, featuring four immunoglobulin (Ig) superfamily domains, is encoded by the LAG3 gene (39). It is expressed on T cells, NK cells, and B cells (40), binding to major histocompatibility complex II (MHC-II), liver and lymph node sinusoidal endothelial cell C-type lectin, and fibrinogen-like protein 1, all of which are found on antigen-presenting cells and tumor cells. Liver and lymph node sinusoidal endothelial cell C-type lectin, a C-lectin receptor on the cell surface, and MHC-II display the highest affinity for LAG-3 (27). Like other immune checkpoints, LAG-3 inhibits T cell proliferation, cytotoxicity, and homeostasis, hindering immune responses against tumor cells and foreign invaders (41–43). Elevated expression of LAG-3 alongside PD-1 is associated with a poor prognosis for patients with ccRCC (44).

Relatlimab, a humanized IgG4 monoclonal antibody, specifically targets the LAG-3 protein and is currently undergoing Phase II clinical trials for ccRCC. Recent findings suggest that monotherapy with anti-LAG-3 or its combination with anti-PD-1 shows promise for patients with LAG-3+ ccRCC who are refractory to anti-PD-1 therapy. Administered intravenously, Relatlimab may cause adverse reactions such as fatigue, rash, and arthralgia. Additionally, its combination with other immune checkpoint inhibitors, such as Nivolumab, is being investigated to improve therapeutic efficacy against RCC (44).

##### 2.1.2.4 T cell immunoglobulin and Mucin domain 3 inhibitors

T cell Immunoglobulin and Mucin domain 3 (TIM-3), also known as Hepatitis A Virus Cellular Receptor 2 (HAVCR2), is a transmembrane immune checkpoint receptor involved in tumor-driven immune suppression. First identified by Kuchroo et al. in 2002, the *HAVCR2* gene encodes this protein (45), initially observed on CD4^+^ and CD8^+^ T cells. Subsequent studies confirmed its expression on T helper 17 cells (46), Regulatory T cells (47), and innate immune cells such as DCs, NK cells, and monocytes (48). Structurally, TIM-3 comprises a membrane-distal single variable Ig domain, a glycosylated mucin domain, and an intracellular stem (49). TIM-3 interacts with multiple ligands, including galectin-9, high mobility group box 1 protein, phosphatidylserine, and carcinoembryonic antigen-related cell adhesion molecule 1. Binding between TIM-3 and its ligands results in NK and T cell dysfunction, ultimately leading to immune suppression in cancer and viral infections (50, 51). TIM-3 has been implicated in several malignancies, such as RCC, melanoma, and gastric cancer (52). However, its precise role in ccRCC remains unclear (53). TIM-3 blockade alone shows limited efficacy, necessitating its combination with other immune checkpoint inhibitors, such as anti-PD-1 antibodies, to enhance therapeutic outcomes (54).

In hematological malignancies, TIM-3 is found on CD8^+^ T cells in myelodysplastic syndrome, indicating its broader involvement beyond solid tumor immunity exhaustion (55). Sabatolimab, a pioneering immuno-myeloid agent targeting the TIM-3 transmembrane protein, is a humanized monoclonal antibody (IgG4). It is currently undergoing clinical trials for treating immunogenic cancers (56, 57).

##### 2.1.2.5 T cell immunoreceptor with immunoglobulin and ITIM domains inhibitors

T cell Immunoreceptor with Immunoglobulin and ITIM domains (TIGIT), also known as WUCAM or VSTM3, is a transmembrane protein discovered in 2009. It belongs to the CD28 immunoglobulin superfamily and is encoded by the *TIGIT* gene. This 244-amino-acid protein (58) consists of an immunoglobulin domain, a transmembrane region, and an inhibitory cytoplasmic tail (59, 60). TIGIT is expressed on tumor-reactive T cells, NK cells, DCs, and macrophages, where it impedes antitumor activation and immune functionality (52, 61, 62). TIGIT binds to ligands such as CD155, CD112, and CD113. Ligand engagement triggers the phosphorylation of intracellular domains, leading to the recruitment of protein Growth factor receptor-bound protein 2 and inhibition of immune active SH2 domain-containing inositol 5′-phosphatase 1, Phosphatidylinositol 3-kinase (PI3K), and Mitogen-Activated Protein Kinase (MAPK) pathways (52, 60, 63). TIGIT expression varies across cancers and is generally low in RCC. However, tumor-infiltrating NK cells from metastatic lymph node patients exhibit elevated TIGIT expression, indicating a role in immune suppression (52).

Tiragolumab, a human IgG4κ monoclonal antibody targeting TIGIT, is currently under clinical evaluation (Phase II) for metastatic RCC. TIGIT has emerged as a promising therapeutic target for cancer immunomodulation, particularly when combined with PD-1 inhibitors. Preclinical studies have demonstrated that blocking both TIGIT and PD-1 significantly enhances tumor-specific T cell proliferation, degranulation, and cytokine production (64).The adverse reactions to Tiragolumab are similar to those of other ICIs, including fatigue, chills, and nausea (59, 65).

#### 2.1.3 Adoptive cell therapy

##### 2.1.3.1 Non-gene-modified cell therapies

###### 2.1.3.1.1 Cytokine-Induced Killer cells

Cytokine-Induced Killer (CIK) cells are a heterogeneous population derived from peripheral or umbilical cord blood mononuclear precursors after stimulation with Interferon-gamma (IFN-γ), anti-CD3 monoclonal antibodies, and IL-2. This cell subset includes CD3^+^CD56^+^ NK-like cells, CD3^+^CD56^−^ T lymphocytes, and CD3^−^CD56^+^ NK cells (66). CIK cells exhibit robust preclinical anti-tumor activity independent of major histocompatibility complex (MHC) or T cell receptor (TCR) specificity (66, 67). They express a diverse TCR repertoire and NK-associated markers such as Natural Killer Group 2 Member D, DNAX Accessory Molecule-1, and Natural Killer Cell p30-Activating Receptor (67), with Natural Killer Group 2 Member D engagement playing a pivotal role in their cytotoxic response against neoplastic cells (68).

In a prospective study comparing autologous CIK therapy to subcutaneous IL-2/Interferon-alpha (IFNα)-2a in 148 patients with metastatic ccRCC (69). CIK-treated patients demonstrated superior outcomes, including higher objective response rate (ORR) (53% vs. 27%), improved 3-year progression-free survival (PFS) (18% vs. 12%, p=0.031), and extended median overall survival (OS) (46 vs. 19 months, p<0.001). Subsequently, a randomized study involving 20 post-radical nephrectomy stage I/II patients contrasted autologous CIKs with investigator-selected treatments. Two weeks post-infusion, CIK-treated patients showed enhanced CD3^+^, CD3^+^CD8^+^, and CD3^+^CD56^+^ populations in peripheral blood (70). The CIK group also demonstrated significantly prolonged median PFS (32.2 vs. 21.6 months, p=0.032). In a separate study of 29 metastatic RCC (mRCC) patients, CIK therapy achieved a modest ORR of 13.8%, with elevated circulating myeloid-derived suppressor cells (MDSCs) correlating with poor outcomes, emphasizing the role of the tumor microenvironment (TME) in disease prognosis (71). Other mRCC CIK trials documented low toxicity and CD3^+^CD56^+^ cell expansion *in vivo*, yet definitive clinical benefits were obscured by study limitations including small sample sizes and heterogeneity (72, 73).

Incorporating DC vaccination to present tumor antigens, secrete cytokines, and engage in direct cell contact has shown promise in enhancing CIK activation and cytotoxicity (74). A retrospective study of 410 post-surgical mRCC patients revealed that DC-CIK therapy significantly outperformed IFNα, achieving a higher 3-year OS (96% vs. 83%, p<0.01) (75). Another randomized trial comparing autologous DC-CIK+IFNα vs. no adjuvant post-RCC surgery showed reduced recurrence and metastasis rates (p<0.01) (76). Similar findings were observed in a study where DC-CIK therapy led to a marked decline in post-surgery relapse (p = 0.0418) and superior 3-year DFS rates (96.7% vs. 57.7%) compared to no adjuvant therapy (75).

CIK cells are attractive therapeutic candidates due to their manufacturability and low toxicity. Future strategies focus on combining CIK cells with DC vaccines, TKIs, and ICIs to overcome the immunosuppressive TME and enhance therapeutic efficacy. A global registry for CIK cells is envisioned to establish benchmarks for future research endeavors (77).

###### 2.1.3.1.2 Tumor infiltrating lymphocytes

Tumor-infiltrating lymphocytes (TILs) represent a promising adoptive cell therapy, especially for immunogenic tumors like metastatic melanoma, where clinical studies report an ORR of 49-72% and complete response (CR) rates of 10-20%, with approximately 40% achieving durable responses (78, 79). TILs are polyclonal T cells propagated ex vivo from patient tumor samples and re-infused after lymphodepleting chemotherapy, typically cyclophosphamide and fludarabine, to enhance *in vivo* expansion (78, 79).

Although promising in melanoma, TILs therapies have shown limited success in RCC. An early phase 3 clinical trial comparing CD8+ TILs combined with IL2 versus IL2 monotherapy in post-radical nephrectomy mRCC patients reported a modest ORR of 9.9%, compared to 11.4% in the IL2-only group (80). The trial was prematurely terminated due to insufficient efficacy, possibly attributed to diminished TIL viability and functional defects resulting from prolonged ex vivo culturing.

Recent advancements in TILs manufacturing, particularly the Rapid Expansion Protocol (REP) initially developed for melanoma, are being tested in mRCC. Despite successful TIL expansion using anti-CD3, high-dose IL-2, and irradiated allogeneic feeder cells, the products often exhibit functional defects, including poor cytotoxicity and reduced cytokine secretion (81). This may result from the limited T-cell recognition of tumor-specific antigens or manufacturing-induced quality decline (82).

Single-cell RNA sequencing analysis of RCC-derived TILs before and after REP revealed preferential expansion of CD4+ T cells, reduced T-cell diversity, and stagnation of exhausted T-cell clones (83). Emerging methods, such as Dynabeads-based manufacturing, have demonstrated improved TIL expansion and functionality (84). Achieving polyclonal, tumor-specific T-cell diversity and reactivity remains pivotal for advancing TIL therapies targeting RCC.

##### 2.1.3.2 Gene-modified adoptive cell therapies

Chimeric Antigen Receptor T-Cells (CAR-Ts) and NK Cells (CAR-NKs).

Chimeric Antigen Receptor T-Cells (CAR-Ts) are T lymphocytes genetically reprogrammed to recognize and kill tumor cells independently of HLA presentation. This contrasts with endogenous T cells, which require peptide-HLA complexes for activation. By circumventing HLA restriction, CAR-T cells offer broader applicability and can target a wide range of surface antigens. Structurally, CARs are modular receptors composed of an extracellular antigen-binding domain, a hinge/spacer, a transmembrane domain, and intracellular signaling motifs (85). The antigen-binding domain typically derives from a single-chain variable fragment of tumor-specific antibodies, though nanobodies and ligands have also shown efficacy (86, 87).

The hinge and transmembrane domains, sourced from proteins like CD8 or CD28, modulate flexibility and anchoring. Intracellular domains usually consist of CD3ζ and one or more co-stimulatory motifs, enabling potent T cell activation and cytokine secretion. Unlike natural T cells, CAR-Ts integrate signal 1 and signal 2 into a single receptor, facilitating autonomous tumor engagement (85).

Five CAR generations have evolved since their 1987 inception (88). First-generation CARs included only CD3ζ and lacked persistence. Second- and third-generation CARs introduced co-stimulatory domains, improving cytotoxicity and expansion (89). More advanced fourth-generation CARs, known as T cell Redirected for Universal Cytokine Killing, are engineered to secrete immunostimulatory cytokines like IL-12, IL-15, or IL-18, thereby enhancing anti-tumor efficacy and modulating the tumor microenvironment (90). Fifth-generation CARs incorporate cytokine receptor signaling to promote persistence and mimic physiological T cell signaling (91).

To enhance safety, “suicide switches” (e.g., inducible caspase-9) enable pharmacologic CAR-T ablation in AE (92). CAR-T production involves leukapheresis, gene modification (commonly via viral vectors), and expansion. While viral vectors ensure stable gene integration, they carry rare risks of insertional mutagenesis and secondary malignancies (93–95). Alternatives such as CRISPR/Cas9, TALENs, and transposon systems offer non-viral solutions with enhanced safety profiles (96).

While CAR-T cells have shown significant success in hematologic malignancies, their application in solid tumors like RCC faces unique challenges (97). Solid tumors are shielded by physical and immunosuppressive barriers (e.g., hypoxia, acidic pH, Tregs), limiting CAR-T infiltration and function. Tumor antigen heterogeneity and off-tumor toxicity (due to shared antigen expression with healthy tissues) further complicate treatment.

Unlike T cells, NK cells naturally eliminate tumor cells without HLA recognition (98). Allogeneic NK cell therapies have been explored, though clinical outcomes have been modest thus far. Chimeric Antigen Receptor NK Cells (CAR-NK) are now under early-stage clinical evaluation (99). Their inherent lack of HLA restriction makes them attractive candidates for off-the-shelf manufacturing, though protocols for efficient expansion and gene modification still require optimization. Similarly, macrophages, which are often recruited into tumor tissues and play key roles in shaping the immune microenvironment, have emerged as novel platforms for CAR engineering (100, 101).

In RCC, multiple tumor-associated antigens have been identified for CAR-targeting, including Carboxy-Anhydrase IX (CAIX), CD70, AXL receptor tyrosine kinase (AXL), Receptor tyrosine kinase-like orphan receptor 2 (ROR2), DnaJ heat shock protein family (Hsp40) member B8 (DNAJB8), Mucin 1, C-Mesenchymal-Epithelial Transition Factor (c-Met), and Epidermal Growth Factor Receptor (EGFR). CAR-Ts and CAR-NKs therapies against these targets are under various stages of development, with some already in clinical trials (**Table 2**).

**Table 2:** Selected pivotal CAR Therapy Clinical Trials for RCC.

Target protein	Description	Clinical trial	Phase	Response rate	Status
CAR T therapy					
CD70	Allogeneic ALLO-316	NCT04696731	1	No study results	Recruiting
CD70	CTX130	NCT04438083	1	No study results	Terminated
CD70	CD70-targeting CAR-T cells	NCT06010875 NCT05468190	1	No study results	Recruiting
CD70	CD70-targeted CAR-T	NCT05420545 NCT05420519	1	No study results	Recruiting
CD70	CD70-targeted CAR-T	NCT05795595	1	No study results	Recruiting
ROR2	Autologous CCT301-38 or CCT 301-59 T cells	NCT03393936	1/2	No study results	Terminated
VEGFR2	Anti-VEGFR2 gene modified tumour white blood cells	NCT01218867	1/2	No study results	Terminated
c-MET	Autologous CAR-T/TCR-T cell immunotherapy	NCT03638206	1/2	No study results	Unknown
CAIX	CAIX-targeted CAR-T Cells	NCT04969354	1	No study results	Recruiting
Mucin 1 cell surface associated C terminal	P-MUC1C-ALLO1 allogeneic CAR-T cells	NCT05239143	1	No study results	Recruiting
Human leukocyte antigen (HLA-G)	Autologous HLA-G-Targeted CAR-T Cells IVS-3001	NCT05672459	1/2	No study results	Recruiting
CAR NK cell therapy					
CD70	CAR.70-engineered IL15-transduced Cord Blood-derived NK Cells in Conjunction with Lymphodepleting Chemotherapy	NCT05703854	1/2	No study results	Recruiting
CAR PBL therapy					
CD70	PBL Transduced with a CD70-Binding CAR	NCT02830724	1/2	No study results	Recruiting

###### 2.1.3.2.1 CAIX

CAIX is an enzyme frequently upregulated in hypoxic solid tumors, notably mRCC, making it an early target for CAR-T therapy in these malignancies (102). In a Phase I trial involving 12 patients, first-generation autologous anti-CAIX CAR-T cells were administered without lymphodepletion preconditioning. No clinical responses were observed, and significant toxicities, including liver enzyme disturbances, were reported (103).

Subsequent research has focused on enhancing the efficacy and safety of anti-CAIX CAR-T therapies. A self-inactivating bi-cistronic CAR-T targeting CAIX, fused with a CD28ζ endodomain and designed to locally release anti-PD-L1 antibodies at RCC sites, demonstrated heightened antitumor efficacy and reduced T-cell exhaustion markers in a humanized mouse model of CAIX^+^PD-L1^+^RCC (104). This suggests that modulating the TME is pivotal in mRCC prognosis (105).

To address antigenic heterogeneity, dual-targeting strategies have been explored. A dual-targeted fine-tuned immune-restoring CAR-T for ccRCC, aimed at both CAIX and CD70, and engineered to co-release immune checkpoint inhibitors, is currently under preclinical assessment (106).

Combining anti-CAIX CAR-T products with TKIs like sunitinib has shown promise (107). Sunitinib is known to augment IFNγ-producing T-cells while diminishing regulatory T cells and MDSCs (108, 109). In a humanized RCC mouse model, the combination of sunitinib and anti-CAIX CAR-Ts significantly reduced tumor load, outperforming either monotherapy (107).

Regarding CAR-modified NK cells, third-generation anti-CAIX CAR-modified NK92 cells, combined with bortezomib, reduced tumor burden in immunocompromised RCC xenograft mice (110). Bortezomib is believed to augment NK-mediated antitumor responses (111).

These findings underscore the potential of targeting CAIX in RCC using CAR-T and CAR-NK cell therapies, while highlighting the importance of addressing associated toxicities and the immunosuppressive tumor microenvironment.

###### 2.1.3.2.2 CD70

CD70 is a transmembrane glycoprotein that interacts with CD27 receptors on T cells, promoting the generation of effector and memory T cells (112). Its expression is notable in activated T and B lymphocytes, as well as mature dendritic cells (113). Importantly, CD70 is prominently expressed in RCC, making it a compelling target for CAR-T therapy (114).

The CTX130 Phase I multicenter trial evaluated an allogeneic CD70-targeting CAR-T therapy in 16 patients with relapsed or refractory ccRCC. Preclinical studies demonstrated favorable proliferation and cytotoxicity profiles, with complete regression of RCC xenograft tumors. In the clinical setting, patients received escalating doses of CTX130 without encountering dose-limiting toxicities. Disease control was achieved in 81.3% of patients, and one patient remained in a durable complete response at three years. The study also introduced CTX131, a next-generation CAR-T construct with synergistic potency enhancements, which demonstrated improved expansion and efficacy in preclinical models. These findings establish proof of concept for CD70-targeted allogeneic CAR-T therapies in ccRCC and other CD70-expressing malignancies (115).

In contrast, the TRAVERSE study assessed ALLO316, an allogeneic *Transcription Activator–Like Effector Nuclease* gene-edited anti-CD70 CAR-T product, in 17 patients with pretreated advanced or metastatic ccRCC. To mitigate the risk of graft-versus-host disease, the T-cell receptor alpha constant gene was deleted, and CD52 was ablated to enhance lymphodepletion using ALLO647 (a humanized anti-CD52 antibody) in combination with fludarabine and cyclophosphamide. Cytokine Release Syndrome occurred in 65% of patients, including one case of grade 3 severity. The disease control rate reached 71%, peaking at 100% in CD70-positive subsets.

Additionally, CRISPR/Cas9-edited CAT-248 CAR-NK cells have been developed to target CD70. These cells are engineered to prevent self-targeting by deleting endogenous CD70 and secrete interleukin-15 (IL-15) to enhance persistence. In preclinical studies, CAT-248 demonstrated efficacy *in vitro* and in xenograft RCC models, significantly reducing tumor burden by more than 99%.

These findings underscore the potential of CD70-targeted CAR-T and CAR-NK therapies in treating CD70-expressing malignancies, particularly RCC.

###### 2.1.3.2.3 AXL & ROR2

AXL, a member of the TAM family tyrosine kinase receptors, is frequently overexpressed in various solid tumors, including RCC (116), where it interacts with its high-affinity ligand growth arrest-specific protein 6 to promote proliferation, survival, angiogenesis, and invasion (117). Similarly, ROR2, an orphan tyrosine kinase receptor critical during embryogenesis, exhibits limited expression in adult tissues but is aberrantly upregulated in RCC, where it contributes to enhanced tumor growth, migration, and invasiveness (118). Given their roles in tumor progression, both AXL and ROR2 have emerged as promising targets for CAR-T therapies. A Phase I/II clinical trial investigated the safety and efficacy of ROR2-targeted CAR-T (CCT301-59) and AXL-targeted CAR-T (CCT301-38) therapies in patients with refractory or relapsed metastatic RCC.

###### 2.1.3.2.4 DNAJB8

DNAJB8, a cancer-testis antigen, has emerged as a potential therapeutic target due to its expression in cancer stem/initiating cells and implication in tumorigenicity in RCC and osteosarcoma. Recent developments in CAR-T cell therapy have focused on second-generation constructs utilizing the B10 binder (B10-CAR), specifically targeting Human Leukocyte Antigen(HLA)-A*24:02/DNAJB8 peptide complexes on RCC cells (119). These engineered cells demonstrated robust antigen-specific and HLA-mediated activation, inducing IFN-γ secretion *in vitro* when exposed to RCC cell lines and significantly reducing tumor burden *in vivo*.

Although these findings highlight the therapeutic potential of targeting cancer stem-like cell antigens, further investigations are required to validate this approach and optimize its clinical efficacy.

###### 2.1.3.2.5 Mucin 1

Mucin 1, a transmembrane glycoprotein normally expressed on epithelial surfaces, plays a protective role in healthy tissues but frequently undergoes aberrant glycosylation and overexpression in various malignancies, including RCC (120). An allogeneic CAR-T therapy targeting the Mucin 1 C-terminal antigen utilizes Cas-CLOVER gene editing to eliminate TCR and MHC-I expression, thereby mitigating the risk of graft-versus-host disease and immune rejection. This therapy is currently being evaluated in a Phase I clinical trial involving patients with advanced or metastatic epithelial tumors, including RCC. Further investigations are required to assess its efficacy and safety in this patient population.

###### 2.1.3.2.6 c-met

c-Met, a receptor tyrosine kinase involved in tumor cell migration, proliferation, and invasion, is overexpressed in approximately 97% of papillary RCC cases while being largely absent in healthy renal tissue (121). A third-generation CAR-T cell therapy targeting c-Met, incorporating CD28, 4-1BB, and CD3ζ co-stimulatory domains, demonstrated antitumor efficacy in an orthotopic RCC mouse model, where tumor growth inhibition was observed in 60% of cases. Histological analysis confirmed the infiltration of CAR+ and CD8^+^ T cells into the tumor microenvironment. Furthermore, when combined with Axitinib, a tyrosine kinase inhibitor, the therapy produced a synergistic antitumor effect, warranting further investigation in clinical studies (121).

###### 2.1.3.2.7 EGFR

EGFR Specific CAR-NK92 construct has been evaluated in combination with Cabozantinib, a tyrosine kinase inhibitor, demonstrating significant antigen-specific activation and enhanced antitumor efficacy in both *in vitro* and *in vivo* preclinical models (122). Cabozantinib potentiates CAR-NK92 activity by upregulating EGFR expression and downregulating PD-L1 on tumor cells, thereby improving immune cell infiltration and reducing immune suppression within the tumor microenvironment (122). This synergistic interaction highlights the potential of combining CAR-NK92 therapy with Cabozantinib for targeting EGFR-positive solid tumors, particularly in RCC.

#### 2.1.4 DCs

DCs, as the most potent antigen-presenting cells, play a pivotal role in initiating T-cell activation by cross-presenting tumor antigens via MHC-I and MHC-II molecules, thereby inducing durable immunological memory against pathogens and tumors (123, 124). Harnessing DC-mediated antitumor responses has been a central focus of cell-based cancer immunotherapies, with strategies ranging from loading DCs with tumor lysates, proteins, and peptides to genetically engineered vectors encoding tumor-specific neoantigens (125–129). Clinical studies have demonstrated the safety and partial efficacy of antigen-loaded DC vaccines, albeit with limited response rates (10-20% in most trials), highlighting the need for improved therapeutic strategies (130–133). Significant barriers, including tumor-mediated immune suppression (e.g., via IL-10 and TGF-β), downregulation of antigen expression, regulatory T-cell dominance, and robust checkpoint pathways (e.g., PD-1/PD-L1), impede the full potential of DC-based therapies (134, 135). To overcome these challenges, researchers are exploring combinatorial approaches, such as integrating DC vaccines with immune checkpoint inhibitors, cytokine modulation (e.g., IL-12 and IFN-α), and neoantigen-specific targeting, aiming to achieve more sustained clinical outcomes and broader patient benefits (136).

#### 2.1.5 Vaccines

Cancer vaccines represent a promising immunotherapeutic strategy designed to stimulate the immune system to recognize and eliminate malignant cells (137, 138). Unlike traditional prophylactic vaccines that prevent virus-associated cancers, therapeutic cancer vaccines aim to enhance anti-tumor immunity in patients with established malignancies (139, 140). These vaccines introduce tumor-associated antigens or neoantigens to activate DCs, which in turn prime cytotoxic T lymphocytes to mount a targeted immune response against tumor cells (141, 142). While initial attempts using autologous tumor cell vaccines did not demonstrate significant clinical efficacy in phase III trials (143, 144), recent advances have focused on combining vaccines with ICIs to amplify immune responses. This synergistic approach aims to overcome tumor-induced immune suppression and improve therapeutic outcomes.

One promising strategy is NeoVax, a personalized neoantigen vaccine derived from tumor DNA sequencing. By incorporating synthetic neoantigens alongside the toll-like receptor agonist polyinosinic-polycytidylic acid stabilized with poly-L-lysine and carboxymethylcellulose, NeoVax has been shown to elicit durable immune responses (145). A phase I study in high-risk melanoma patients demonstrated its ability to induce sustained T-cell immunity (146). Building on these findings, a phase I trial (NCT02950766) is currently evaluating the combination of NeoVax and ipilimumab in RCC.

Despite encouraging preliminary results, challenges remain in fully harnessing the therapeutic potential of cancer vaccines for RCC. The immunosuppressive tumor microenvironment and tumor heterogeneity present significant hurdles to achieving sustained clinical benefit. While no RCC-specific vaccines have received regulatory approval, ongoing research is focused on improving vaccine formulations, identifying optimal target antigens, and integrating vaccines with existing treatment strategies to enhance efficacy. Continued clinical trials will be essential in defining the role of vaccine-based immunotherapy in RCC management.

#### 2.1.6 Summary of immunotherapy for RCC

The landscape of RCC treatment has been transformed by immunotherapy, with ICIs, adoptive cell therapies, and cancer vaccines demonstrating significant clinical potential. By disrupting inhibitory pathways and enhancing immune activation, these approaches have improved patient outcomes, particularly in advanced RCC. However, despite these advancements, immunotherapy alone is often insufficient to achieve durable tumor control. Given the molecular complexity of RCC, targeted therapies remain essential, directly inhibiting oncogenic signaling pathways that drive tumor progression. The integration of immunotherapy with targeted agents represents a promising strategy, leveraging the strengths of both modalities to optimize treatment efficacy and overcome resistance mechanisms.

## 3 Targeted therapy in RCC

### 3.1 Targeted therapy origins

**Figure 2:**
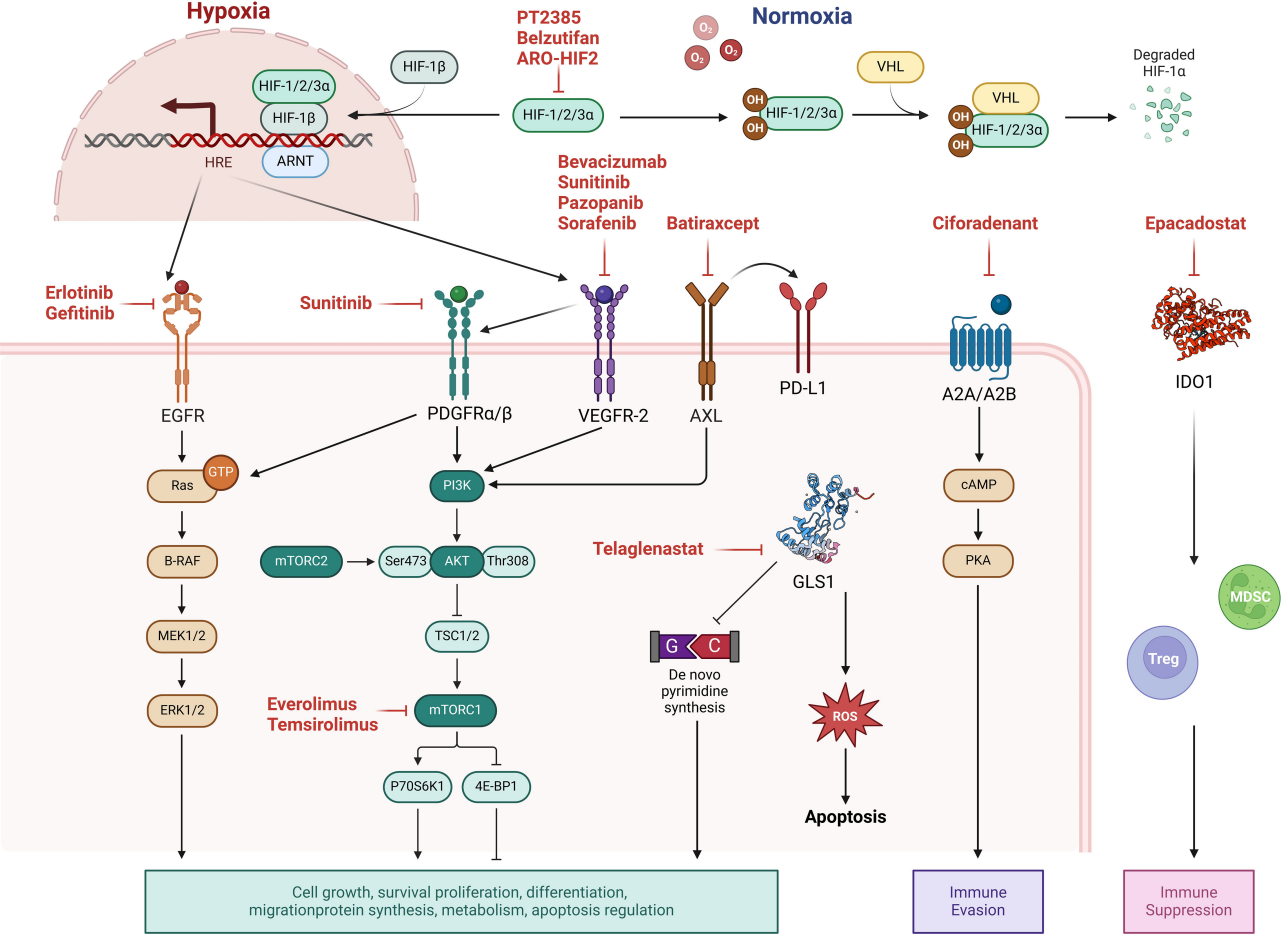
Molecular targets and pathway modulations in renal cell carcinoma (RCC) therapy. Current targeted therapies for RCC focus on key oncogenic drivers: (1) Hypoxia-inducible factor 2α (HIF2α) inhibition in clear cell RCC (ccRCC) to disrupt hypoxia response signaling; (2) Growth factor signaling blockade targeting vascular endothelial growth factor (VEGF), platelet-derived growth factor (PDGF), and epidermal growth factor (EGF) pathways; (3) mTOR pathway modulation through inhibition of mechanistic target of rapamycin (mTOR); (4) Metabolic reprogramming interventions including Inhibition of glutaminase 1 (GLS1); (5) Receptor tyrosine kinase inhibition (e.g., AXL kinase blockade); and (6) Adenosine receptor antagonism to counteract immunosuppressive tumor microenvironments. These strategies collectively aim to interfere with tumor survival, proliferation, and microenvironmental adaptation.

The advent of targeted therapy emerged from breakthroughs in understanding cancer molecular mechanisms in the late 20th century. The approval of imatinib (Gleevec) in the late 1990s, the first successful TKI, revolutionized cancer treatment by dramatically improving outcomes for chronic myeloid leukemia through the specific inhibition of Breakpoint Cluster Region-Abelson (147). This milestone spurred the development of targeted therapies across various cancers, including RCC. In 2005, the approval of sorafenib marked the introduction of targeted treatment for RCC, paving the way for multi-targeted TKIs (148). Currently, the targeted therapies for RCC mainly focus on key oncogenic drivers, as is shown in **Figure 2**.

ccRCC, the most prevalent RCC subtype, frequently harbors mutations in the tumor suppressor gene von Hippel-Lindau (*VHL*). VHL loss leads to aberrant stabilization and overexpression of hypoxia-inducible factor 2 alpha (HIF2α), which dysregulates multiple tumorigenic pathways (149). HIF2α promotes angiogenesis by upregulating vascular endothelial growth factor (VEGF) and platelet-derived growth factor (PDGF), enhances cell proliferation via cyclin D1 and glucose metabolism through glucose transporter 1, and drives tumor invasion and metastasis via stromal cell-derived factor 1 and its receptor C-X-C motif chemokine receptor 4 (150). Additionally, HIF2α attenuates EGFR endocytosis, sustaining EGFR-mediated signaling and contributing to tumor growth and progression (151, 152).

In RCC, the mTOR pathway is frequently hyperactivated, often due to *VHL* mutations or dysregulated upstream signaling (9). This aberrant activation promotes downstream targets, including S6 kinase and eukaryotic translation initiation factor 4E-binding protein 1, enhancing protein synthesis and cellular proliferation (10). The intricate interconnections between the VHL-HIF2α pathway and mTOR signaling underscore their collective role in RCC pathogenesis, offering multiple potential therapeutic targets. Consequently, therapeutic interventions targeting these pathways continue to be a focus of ongoing research and clinical trials (11, 12) (**Table 3** **).**

**Table 3:** Selected pivotal studies of targeted therapies approved as monotherapies for the treatment of RCC.

Target protein	Experimental arm	Clinical trial	Phase	Response rate	Status
mTOR	Temsirolimus	NCT00065468	3	OS 10.9months	Completed
mTOR	Everolimus	NCT00410124	3	PFS 4.90months	Completed
VEGFR, PDGFR, c-Raf	Sorafenib	NCT00073307	3	OS 542 days	Completed
VEGFR, PDGFR, c-Kit, RET	Sunitinib	NCT00083889	3	PFS 48.3weeks	Completed
VEGFR, PDGFR, FGFR, c-Kit	Pazopanib	NCT00334282	3	OS 22.9months	Completed
VEGFR, PDGFR, c-Kit	Axitinib	NCT00678392	3	PFS 6.7months	Completed
VEGFR, c-Met, AXL	Cabozantinib	NCT01865747	3	OS 21.4months, PFS 7.4months	Completed
VEGFR, c-Met, AXL	Cabozantinib	NCT01835158	2	PFS 8.2months	Completed
mTOR	Temsirolimus	NCT00065468	3	OS 10.9months, PFS 5.5months	Completed

#### 3.1.1 HIF2α inhibition in ccRCC

The *VHL* tumor suppressor gene, located on chromosome 3p, plays a central role in the pathogenesis of ccRCC. Inactivating mutations in both alleles of VHL represent the most prevalent genetic alterations in ccRCC, observed in both sporadic and hereditary cases (153). VHL disease, an autosomal dominant hereditary cancer syndrome, stems from germline VHL mutations, conferring a 70% lifetime risk for RCC, along with increased susceptibility to tumors such as hemangioblastomas and paragangliomas (154).

The VHL protein (pVHL) functions as a key component of the E3 ubiquitin ligase complex, which regulates the degradation of hypoxia-inducible factors (HIFs), including hypoxia-inducible factor 1 alpha (HIF1α), hypoxia-inducible factor 2 alpha (HIF2α), and hypoxia-inducible factor 3 alpha (HIF3α) (155). Among these, HIF2α has emerged as a critical driver of ccRCC progression (153). Under normoxic conditions, the pVHL/E3 ubiquitin ligase complex targets HIF1α and HIF2α for proteasomal degradation (156). VHL inactivation leads to the accumulation of HIFα subunits, which dimerize with HIFβ to form an active transcriptional complex. This complex induces the expression of hypoxia-responsive genes such as VEGF, PDGF-β, and erythropoietin, contributing to ccRCC’s characteristic hypervascularity and paraneoplastic erythrocytosis (154). Clinical trials have been conducted to evaluate the efficacy and safety of enrolled HIF-2α inhibitors for RCC (**Table 4** **).**

**Table 4:** Selected pivotal HIF-2α inhibitors Clinical Trials for RCC.

Target Protein	Experimental arm	Clinical trial	Phase	Status
HIF-2α	Belzutifan (PT2385)	NCT02293980	1	Active, not recruiting
HIF-2α and PD-1	Belzutifan (PT2385) + nivolumab	NCT02293980	1	Active, not recruiting
HIF-2α	Belzutifan (MK-6482)	NCT02974738	1	Active, not recruiting
HIF-2α	Belzutifan (MK-6482)	NCT03401788	2	Active, not recruiting
HIF-2α	Belzutifan (PT2385)	NCT03108066	2	Completed
HIF-2α and VEGFR, c-Met, AXL	Belzutifan (PT2977, MK-6482) + cabozantinib	NCT03634540	2	Active, not recruiting
HIF-2α	Belzutifan (MK-6482)	NCT04195750	3	Active, not recruiting
HIF-2α	ARO-HIF2	NCT04169711	1	Completed

Initially considered an undruggable target due to the absence of a clear ligand-binding domain, HIF2α became a viable therapeutic target with the identification of a small binding site within its Per-Arnt-Sim B domain. This discovery led to the development of PT2385, the first HIF2α-specific inhibitor (157, 158).

A Phase I dose-escalation trial involving 51 pretreated metastatic ccRCC patients demonstrated PT2385’s favorable safety profile, with anemia, peripheral edema, and fatigue as the most common adverse effects. Partial and complete responses were observed in 12% and 2% of patients, respectively, while 52% achieved disease stabilization (159). Pharmacokinetic and pharmacodynamic analyses identified 800 mg twice daily as the optimal dose for Phase II trials. Notably, PT2385 rapidly suppressed HIF2α-mediated erythropoietin expression, confirming target engagement and biological activity (160).

Despite these promising findings, PT2385 exhibited variable pharmacokinetics, prompting the development of Belzutifan (MK-6482, formerly PT2977), a second-generation HIF2α inhibitor with improved pharmacokinetic properties (161). Structural modifications, including the replacement of a geminal difluoro group with a cis-vicinal difluoro group, enhanced Belzutifan’s serum availability and binding affinity for HIF2α (162).

The first-in-human Phase I/II study (NCT02974738) evaluated Belzutifan in patients with advanced ccRCC and other solid tumors. Belzutifan achieved an ORR of 24%, with 67% of patients experiencing tumor size reduction. The median PFS was 11.0 months. Anemia was the most common adverse event, primarily managed with EPO replacement without necessitating dose reductions. Hypoxia occurred in 26% of patients but was generally manageable with supplemental oxygen.

Further studies have demonstrated Belzutifan’s efficacy in VHL disease-associated RCC. A Phase II trial (NCT03401788) involving 61 patients reported a 49% ORR and a 98% 12-month PFS. Belzutifan also showed efficacy in non-RCC manifestations, including pancreatic neuroendocrine tumors (77% ORR) and central nervous system hemangioblastomas (30% ORR) (163). Based on these findings, the FDA approved Belzutifan in August 2021 for VHL-associated RCC and other tumors not requiring immediate surgical intervention.

Notably, a recent phase III trial showed that belzutifan significantly improved progression-free survival and objective response rates compared to everolimus in patients with advanced ccRCC previously treated with immune checkpoint inhibitors and antiangiogenic therapies (NCT04195750) (164). The ongoing LITESPARK-001 Phase I study has highlighted Belzutifan’s potential in treating metastatic ccRCC, achieving a 25% ORR over a median 3-year follow-up (165). Combination trials are underway, including Belzutifan with cabozantinib (NCT03634540) (166) and pembrolizumab (NCT05239728) (167), aiming to enhance therapeutic outcomes.

RNA interference (RNAi)-based therapies targeting HIF2α are also emerging as promising strategies (168). ARO-HIF2, an RNAi therapy, demonstrated encouraging interim results in a Phase Ib trial (NCT04169711), with one partial response and stable disease in several patients (169). Several Phase II/III trials assessing HIF2α inhibitors, either solo or combined with ICIs, TKIs, and novel analogs, are ongoing. These findings underscore the potential of novel therapeutic approaches to overcome resistance mechanisms associated with HIF2α inhibition.

The development of HIF2α inhibitors, particularly Belzutifan, marks a significant advancement in ccRCC therapy. Ongoing clinical trials and preclinical studies continue to explore combination strategies and novel therapeutic approaches. As understanding of resistance mechanisms and biomarker development evolves, HIF2α inhibition is poised to remain a cornerstone of targeted therapy for ccRCC.

#### 3.1.2 VEGF signaling pathway in RCC

VEGF is a key driver of angiogenesis and is produced by various cell types, including tumor cells, stromal fibroblasts, inflammatory cells, and endothelial cells (160). VEGF exerts its pro-angiogenic effects primarily by binding to specific receptors on vascular endothelial cells: VEGFR-1, VEGFR-2, and VEGFR-3. Among these, VEGFR-2 is particularly critical for angiogenesis (170). Upon VEGF-A binding, VEGFR-2 undergoes dimerization and activates tyrosine kinases, triggering downstream signaling pathways such as PI3K/Akt and MAPK. This activation promotes endothelial cell survival, proliferation, and neovascularization (171).

In the context of RCC, VEGF and its receptors are markedly upregulated, often in response to hypoxia-induced HIF activation within the tumor microenvironment. The loss of VHL protein, a hallmark of ccRCC, leads to constitutive stabilization of HIF, driving the overexpression of VEGF-A. This upregulated VEGF-A binds to VEGFR-2, accelerating angiogenesis and providing essential nutrients and oxygen to the tumor. Consequently, the VEGF-A/VEGFR-2 axis has emerged as a critical therapeutic target in RCC due to its profound role in stimulating angiogenesis (172).

Moreover, VEGF contributes to immune evasion in RCC by inhibiting the maturation of DCs, impairing T-cell activation, and promoting the recruitment of immunosuppressive regulatory T cells. The interaction between VEGF and immune checkpoints, such as the PD-1/PD-L1 axis, further enhances tumor immune evasion. These insights have prompted the development of combination therapies targeting both angiogenesis and immune checkpoints (173).

A variety of VEGF inhibitors, including monoclonal antibodies and TKIs, have been approved for the treatment of RCC. These agents disrupt VEGF-VEGFR interactions or inhibit receptor tyrosine kinase activity to block angiogenesis and tumor growth. Bevacizumab, a monoclonal antibody targeting VEGF-A, demonstrated improved PFS in phase II clinical trials, although long-term survival benefits remain limited (174). Among TKIs, sunitinib and pazopanib are well-established first-line therapies for mRCC, consistently reducing tumor progression in clinical studies (175). Another TKIs, sorafenib, initially developed as a B-Raf inhibitor, was later found to inhibit VEGFR2 (KDR) in ccRCC due to the pivotal role of pVHL loss. Cabozantinib and lenvatinib are primarily used as second-line treatments; cabozantinib inhibits VEGFRs as well as MET and AXL receptors (176), targeting resistance mechanisms, while lenvatinib concurrently blocks VEGFR and Fibroblast Growth Factor Receptor (FGFR) (177). Recent phase III trials have demonstrated the superiority of both drugs in PFS compared to everolimus in pre-treated patients. Additionally, tivozanib, a selective TKI targeting VEGFR1, VEGFR2, and VEGFR3, has demonstrated significant improvements in median PFS compared to sorafenib in two pivotal phase III clinical trials, underscoring its potential as a promising therapeutic option for advanced RCC (178, 179). Cabozantinib was found to be superior to sunitinib in the front-line setting (180) and was also superior to everolimus in patients previously treated with other TKIs that target VEGF (181, 182). Lenvatinib (in combination with everolimus) was also superior to everolimus alone in previously treated patients with RCC (183). It is important to note that although the perceived greater clinical benefit with cabozantinib or lenvatinib, compared with that of earlier TKIs, has been largely considered to be due to their targeting of resistance pathways to VEGF, the benefit may simply be due to more-potent inhibition of KDR (184).

Complete responses to VEGF inhibitors are rare, with tumors often activating angiogenic escape pathways to restore perfusion (185). Recent TKIs such as cabozantinib and lenvatinib provide dual targeting capabilities to overcome resistance (186). Cabozantinib has shown superiority to sunitinib as a front-line treatment and to everolimus in pre-treated patients. Lenvatinib, combined with everolimus, demonstrated enhanced efficacy compared to everolimus monotherapy (187, 188).

Attempts to target other angiogenic pathways, such as the angiopoietin-Tie axis and the Transforming Growth Factor-beta (TGFβ)-Activin Receptor-Like Kinase 1 (ALK1)-endoglin pathway, have largely been unsuccessful (189, 190). For example, combining trebananib (a peptibody neutralizing Angiopoietin-1 and Angiopoietin-2) with sorafenib did not improve PFS compared to sorafenib alone (191). Similarly, the endoglin inhibitor carotuximab and the ALK1 inhibitor dalantercept failed to show superior efficacy when combined with axitinib compared to axitinib monotherapy (192).

However, it should be noted that inhibition of multiple targets by TKIs can result in various AE, necessitating careful monitoring. Hematologic and hepatic toxicities are particularly significant, and VEGF receptor blockade is associated with hypertension and other cardiovascular complications. Additionally, pazopanib may induce side effects such as hair depigmentation, diarrhea, nausea, anorexia, vomiting, and, in rare cases, posterior reversible encephalopathy syndrome (193).

#### 3.1.3 PDGF signaling pathway in RCC

PDGF comprises four isoforms: PDGF-A, PDGF-B, PDGF-C, and PDGF-D. These isoforms form functional dimers that bind to PDGF receptors (PDGFR), which have two subtypes: PDGFRα and PDGFRβ (194). Upon ligand binding, these receptors dimerize and activate downstream signaling cascades, including the PI3K-AKT and Ras-MAPK pathways (195). This activation promotes processes such as tumor growth, angiogenesis, metastasis, and modulation of the tumor microenvironment in RCC. Therapeutic strategies targeting the PDGF-PDGFR axis have shown promise in inhibiting angiogenesis and cell proliferation, making it a potential avenue for RCC treatment (194).

A key finding from recent studies is the role of VEGF165b, an inhibitory isoform of VEGF-A, in modulating PDGFRβ phosphorylation. This interaction suppresses endothelial cell proliferation and migration, potentially contributing to resistance against antiangiogenic therapies. Evidence from clinical and preclinical studies indicates that concurrent activation of PDGF-B/PDGFRβ and VEGF165b pathways may lead to complex vascular phenotypes and therapeutic resistance (196).

A comprehensive study analyzed the immunohistochemical expression of PDGF-B, and its receptor PDGFRβ in 1,423 prospectively collected tumor samples from patients undergoing radical or partial nephrectomy at a tertiary referral center. Among the 1,091 patients (mean age: 54 years), the majority (88.7%) had ccRCC, followed by papillary (7.5%), chromophobe (2.8%), unclassified (0.4%), and other rare types (0.5%). The findings revealed that PDGFRβ expression was highest in ccRCC, whereas PDGF-B expression were most prominent in papillary RCC. After adjusting for T stage and Fuhrman nuclear grade, multivariate logistic regression analysis demonstrated that PDGF-B (OR = 2.46, P = 0.019) expression were significantly higher in papillary RCC compared to clear cell type. These findings underscore the distinct biological characteristics and angiogenic profiles of RCC subtypes (197).

Another study evaluated the prognostic significance of PDGF-BB expression and differentiated microvascular density in 100 patients with ccRCC using immunohistochemistry on tissue microarrays. The results indicated that higher-grade and more advanced-stage ccRCC tumors exhibited significantly lower PDGF-BB expression and differentiated MVD (P < 0.05). Interestingly, elevated PDGF-BB expression emerged as an independent prognostic factor for improved survival. Incorporating PDGF-BB expression into a clinicopathological model significantly enhanced the predictive accuracy for disease-free survival, increasing Harrell’s concordance index from 0.695 to 0.707. A strong positive correlation was observed between PDGF-BB expression and differentiated microvascular density (r = 0.634, P < 0.001), suggesting that PDGF-BB may play a role in vascular differentiation. These insights suggest that targeting PDGF-BB and its associated pathways may provide therapeutic benefits and enhance current anti-angiogenic strategies for RCC management (198).

PDGF-C plays an essential role in developmental and physiological processes as well as in human diseases. A novel splice variant of PDGF-C, termed PDGF-Cb, encodes an N-terminally truncated protein lacking the signal peptide and Complement C1r/C1s, Uegf, and Bmp1 domain. This variant is co-expressed with PDGF-C in normal tissues. PDGF-Cb is produced as a cytoplasmic protein with a similar intracellular localization to PDGF-C but is not secreted from cells. PDGF-Cb can form heterodimers (PDGF-CCb) with PDGF-C, retaining it within the cytoplasm and targeting it for degradation. In primary RCC tumors, expression of the full-length PDGF-C transcript was elevated, while PDGF-Cb expression remained unchanged. These findings suggest that deregulation of PDGF-C may contribute to RCC tumorigenesis and that PDGF-Cb acts as a dominant-negative molecule modulating PDGF-C secretion (199).

A real-world analysis assessed the effectiveness and safety profile of sunitinib in 702 advanced or metastatic RCC (mRCC) patients from 116 German sites (STAR-TOR registry, NCT00700258). Between 2010 and 2020, sunitinib was administered as first-line (83.5%), second-line (11.7%), or third-line (4.8%) therapy. Clear-cell RCC was the predominant subtype (81.6%). Drug-related AEs were reported in 66.3% of patients, with gastrointestinal disorders being the most common (39.7%). Serious AEs occurred in 13.9% of patients. This study provides critical insights into the real-world outcomes and AE profile of sunitinib in a/mRCC patients (200).

Ball et al. (2007) demonstrated that VEGF-A not only stimulates the expression of PDGFRα and PDGFRβ but also binds directly to both receptor types. This interaction positions VEGF-A as a key regulator for recruiting both endothelial cells and perivascular smooth muscle cells, highlighting its multifaceted role in vascular development. The interplay between PDGFRβ and VEGF pathways promotes endothelial cell migration and proliferation while facilitating the recruitment of perivascular cells, which are essential for stabilizing and maturing blood vessels (201).

In conclusion, targeting the PDGF-B/PDGFRβ axis in combination with VEGF inhibitors may offer a novel therapeutic approach for managing resistance to conventional anti-angiogenic therapies in RCC. Further studies are warranted to elucidate the precise mechanisms underlying these interactions and their clinical implications.

#### 3.1.4 Epidermal Growth Factor signaling pathway in RCC

The Epidermal Growth Factor (EGF) signaling pathway plays a crucial role in the development and progression of RCC. Upon binding to the EGFR, EGF triggers receptor dimerization and autophosphorylation, leading to the activation of downstream pathways such as PI3K/Akt, Ras/Raf/MAPK/ERK Kinase (MEK)/Extracellular Signal-Regulated Kinase (ERK), and Janus Kinase (JAK)/Signal Transducer and Activator of Transcription (STAT). These pathways regulate essential cellular processes, including proliferation, survival, angiogenesis, and metastasis (202). In RCC, particularly the clear-cell subtype, the loss of the VHL gene contributes to upregulated HIF2α activity, which enhances EGFR expression. This upregulation promotes tumor growth and survival, making the EGF/EGFR axis a potential therapeutic target (203).

Despite the established role of EGFR-targeting agents in other cancers, such as non-small cell lung cancer and colorectal cancer, their efficacy in RCC has been limited. Early clinical trials using first-generation EGFR inhibitors, such as erlotinib and gefitinib, showed only modest efficacy in unselected RCC populations (203). This limited success can be attributed to the intrinsic molecular heterogeneity of RCC and compensatory activation of alternative pathways, notably EGF-driven angiogenesis. Efforts to overcome these limitations have led to the investigation of combination therapies targeting both EGF and VEGF pathways.

A notable example involved a phase II trial exploring the combination of erlotinib and bevacizumab, a VEGF inhibitor, in patients with metastatic RCC. The combination demonstrated a modest improvement in progression-free survival compared to erlotinib monotherapy but was associated with significant toxicities, including skin rash and gastrointestinal disturbances (204). Another study evaluated gefitinib in combination with sorafenib, a VEGF-targeting TKI, in refractory RCC patients. Although the combination showed limited clinical benefit, it underscored the importance of patient selection and the need for novel therapeutic strategies (205).

Given the limited efficacy of EGFR inhibitors alone, recent studies have explored their combination with ICIs such as pembrolizumab. Preclinical models suggest that EGFR inhibition may enhance tumor immunogenicity, thereby improving responses to ICIs. Additionally, multi-kinase inhibitors like cabozantinib, which target VEGFR, MET, and AXL, have shown promise in overcoming resistance mechanisms associated with EGFR inhibition (206). The dual inhibition of angiogenesis and EGF signaling offers a promising approach to enhance antitumor efficacy by disrupting key survival pathways in RCC (207).

The use of EGFR inhibitors is associated with various adverse effects. Dermatologic toxicities, particularly acneiform rash, are among the most common side effects and may require corticosteroid treatment. Gastrointestinal disturbances, such as diarrhea and mucositis, are also frequently reported. Hepatotoxicity, characterized by elevated liver enzymes, may necessitate dose adjustments. In rare cases, interstitial lung disease has been observed, requiring immediate intervention. Despite these challenges, the integration of EGFR inhibitors with other targeted therapies holds promise for addressing the complex tumor microenvironment in RCC (208).

Future research efforts are focused on identifying predictive biomarkers to guide patient selection and optimizing combination therapies to maximize therapeutic efficacy. The development of next-generation EGFR inhibitors with improved selectivity and safety profiles is also underway. Understanding the intricate interplay between EGF signaling and other oncogenic pathways remains crucial for developing more effective and durable treatment options for RCC patients.

#### 3.1.5 mTOR signaling pathway in RCC

The mammalian mTOR is an atypical serine/threonine protein kinase that plays a critical role within the PI3K/Akt/mTOR signaling cascade. Activation of this pathway drives the proliferation and invasiveness of renal cancer cells by phosphorylating key downstream targets such as ribosomal protein S6 kinase and eukaryotic translation initiation factor 4E-binding protein 1. These phosphorylation events facilitate protein synthesis and promote cell cycle progression, thereby enhancing the proliferative potential of renal cancer cells (209).

Beyond its role in cell proliferation, mTOR signaling also regulates the epithelial-mesenchymal transition process, granting renal cancer cells enhanced invasive and migratory capacities. Additionally, mTOR signaling protects these cancer cells from apoptotic signals by inhibiting apoptosis-related proteins, thereby promoting their survival and sustained proliferation. Another crucial function of the mTOR pathway is the promotion of angiogenesis within renal cancer tissues through the upregulation of VEGF (210). This neovascularization provides cancer cells with essential nutrients and oxygen, contributing to tumor growth and dissemination.

Given its central role in RCC pathogenesis, inhibiting mTOR activity has emerged as a promising therapeutic strategy. Currently, two mTOR inhibitors, everolimus and temsirolimus, have been approved for the treatment of advanced RCC. Temsirolimus is approved as a first-line therapy, particularly for poor-risk patients, while everolimus is commonly used as a second-line option following the failure of VEGFR inhibitor-based treatments. In a pivotal phase III trial, everolimus demonstrated a trend toward improved recurrence-free survival compared to placebo; however, this benefit was accompanied by an increase in AE, including stomatitis, hypertriglyceridemia, and hyperglycemia (211).

At the molecular level, some ccRCC tumors harbor mutations in genes encoding components of the mTOR pathway, further underscoring the importance of targeting this signaling cascade (212). mTOR functions within two distinct multiprotein complexes, mTORC1 and mTORC2 (213). Rapamycin analogs (‘rapalogs’) such as everolimus and temsirolimus selectively inhibit mTORC1, leading to reduced translation of mRNAs involved in cell survival, proliferation, and angiogenesis (214). Despite relatively low response rates, randomized controlled trials have demonstrated the superiority of everolimus and temsirolimus over interferon-α and placebo, respectively, leading to their regulatory approval for advanced RCC treatment (215, 216).

However, the inhibition of mTORC1 has been associated with the unintended consequence of relieving negative feedback inhibition on mTORC2 (217). This can stabilize hypoxia-inducible factor 2 alpha (HIF2α), thereby enhancing PI3K and Akt-mediated cell survival and proliferation (218–220). Preclinical studies in ccRCC cell lines have suggested that novel agents capable of inhibiting both mTORC1 and mTORC2, as well as PI3K, may be more effective than rapalogs (221, 222). Unfortunately, clinical trials evaluating dual mTORC1/mTORC2 inhibitors (e.g., AZD2014 and sapanisertib) and mTOR/PI3K inhibitors (e.g., apitolisib and BEZ235) encountered significant challenges due to considerable on-target toxicity, such as hyperglycemia (223–225). These adverse effects often necessitated dose reductions, which may have compromised their therapeutic efficacy.

Despite these setbacks, ongoing research continues to explore innovative approaches to improve the efficacy and safety of mTOR-targeted therapies in RCC. Combining mTOR inhibitors with other targeted agents or immune checkpoint inhibitors is being investigated to overcome resistance mechanisms and enhance therapeutic outcomes (210). Recent studies have further revealed that down-regulation of lactotransferrin, a critical protein involved in the innate immune system, promotes metastasis in RCC. Interestingly, this down-regulation has also been associated with increased sensitivity of RCC tumor cells to mTOR inhibitors, suggesting that lactotransferrin expression may serve as a predictive biomarker for therapeutic efficacy (226). Understanding the intricate interactions between mTOR signaling and other oncogenic pathways remains critical for the development of more effective and durable treatments for RCC.

#### 3.1.6 Glutaminase inhibition in RCC

In RCC, particularly in cells deficient in the *VHL* tumor suppressor gene, there is a pronounced reliance on glutamine for metabolic processes. Glutamine serves as a critical substrate for producing key molecules such as citrate and lipids through metabolic pathways that support cellular growth and survival. Inhibition of glutaminase 1 disrupts these pathways by reducing intracellular levels of aspartic acid, thereby inhibiting *de novo* pyrimidine synthesis, which impairs DNA synthesis and cellular proliferation. Moreover, glutaminase 1 inhibition leads to the accumulation of reactive oxygen species, inducing oxidative stress and triggering apoptosis in RCC cells (227).

Telaglenastat (CB-839) is a first-in-class, selective, reversible, and orally active GLS1 inhibitor that has been investigated in several clinical trials for its potential to enhance therapeutic outcomes in RCC. The ENTRATA trial, a randomized, double-blind, phase II study, evaluated the combination of telaglenastat and everolimus (T + E) versus placebo plus everolimus (P + E) in 69 patients with mRCC who had progressed after two or more prior systemic therapies. The trial showed a trend toward improved median PFS with T + E (3.8 months vs. 1.9 months, HR 0.64, p = 0.079). However, grade ≥3 AE (AEs) were more frequent in the T + E group (80%) compared to the P + E group (60%). The most common AEs included anemia (17% vs. 17%), pneumonia (7% vs. 4%), abdominal pain (7% vs. 0%), thrombocytopenia (7% vs. 0%), and fatigue (4% vs. 9%). Despite these toxicities, discontinuation due to AEs was similar between groups (28% vs. 30%) (228).

The subsequent phase II CANTATA trial evaluated telaglenastat in combination with cabozantinib (T + C) versus placebo plus cabozantinib (P + C) in patients with advanced ccRCC who had progressed on prior first- or second-line therapies, including anti-angiogenic or ICI-based regimens. Among 444 randomized patients, no statistically significant difference in PFS was observed between the T + C and P + C groups (median PFS 9.2 months vs. 9.3 months, HR 0.94, p = 0.65). ORR were similar (31% vs. 28%, respectively), and OS data remained immature at the time of analysis. In a prespecified subgroup analysis, patients who had previously received ICI-based therapy showed a trend toward PFS benefit with T + C (11.1 months vs. 9.2 months, HR 0.77) (229).

AE were prevalent in both treatment arms, with grade 3–4 AEs occurring in 71% of patients on T + C and 79% in those on P + C. The most common grade 3–4 AEs included hypertension (17% vs. 18%) and diarrhea (15% vs. 13%). These findings highlight the challenges of combining glutaminase inhibition with other targeted therapies in RCC and underscore the need for better patient selection and toxicity management strategies.

While glutaminase inhibition holds promise as a therapeutic approach in RCC, the modest clinical outcomes observed in current trials suggest that further optimization is necessary. Potential strategies include the development of more potent glutaminase 1 inhibitors, improved combination regimens, and the identification of predictive biomarkers to guide patient selection.

#### 3.1.7 AXL in RCC

AXL is a receptor tyrosine kinase that plays a crucial role in immune regulation and tumor progression. Normally expressed in both immune and non-immune cells, AXL is upregulated in ccRCC and is associated with poor prognosis (230). Elevated AXL levels contribute to immune evasion by increasing PD-L1 expression and promoting the clearance of tumor antigens, thereby limiting effective anti-tumor immune responses (230). Additionally, AXL activation is essential for the PI3K/AKT signaling pathway via VEGF, which may underlie resistance to anti-angiogenic therapies (185).

Targeting AXL has emerged as a promising therapeutic approach in ccRCC. Batiraxcept, a recombinant fusion protein that inhibits AXL by binding to its activating ligand Growth Arrest-Specific 6, is currently being evaluated in a phase 1/2b study in combination with cabozantinib for patients with advanced ccRCC. Interim analysis showed an acceptable safety profile, with the most common AEs being decreased appetite, diarrhea, and fatigue. The ORR was 46%, and a baseline serum soluble AXL (sAXL)/Growth Arrest-Specific 6 ratio of 2.3 or greater was associated with a higher ORR of 67%, suggesting its potential as a predictive biomarker (231).

#### 3.1.8 Adenosine receptor inhibition

Metabolic reprogramming within the TME is another critical mechanism contributing to immune evasion and resistance to ICIs (232). One key pathway involves the breakdown of extracellular Adenosine Triphosphate(ATP) into immunosuppressive adenosine (233). ATP, a metabolite released during pro-inflammatory stimuli, promotes immune responses by activating T cells and inducing cytokine production. However, regulatory mechanisms exist to protect tissues from excessive immune activation. This includes the enzymatic conversion of ATP to adenosine by ectonucleotidases CD39 and CD73 (234).

In the TME, hypoxic conditions upregulate the expression of CD39 and CD73 (235), accelerating the breakdown of ATP into adenosine and leading to its accumulation. Adenosine exerts potent immunosuppressive effects by engaging the adenosine receptors A2A and A2B, which are expressed on various immune cell subsets (236). Hypoxia further increases the expression of A2A and A2B receptors, enhancing cell responsiveness to adenosine and promoting immune evasion (234). Compared to other solid tumors, RCC exhibits higher levels of A2AR and CD73 expression, underscoring the relevance of this pathway in disease progression (237).

Targeting the adenosine pathway has shown promise in clinical trials. Ciforadenant (formerly known as CPI-444), a small molecule A2AR antagonist, has been evaluated in a phase I clinical trial involving patients with advanced refractory RCC. The trial demonstrated clinical responses both as monotherapy and in combination with atezolizumab, an anti-PD-L1 antibody. Importantly, higher baseline levels of adenosine-induced genes in tumor biopsies were associated with tumor regression, suggesting their potential as predictive biomarkers for identifying patients who are likely to benefit from adenosine pathway inhibition (237).

The combination of AXL and A2AR inhibition, alongside ICIs and anti-angiogenic therapies, holds promise for overcoming immune resistance and enhancing treatment outcomes in RCC. Continued research and clinical validation are needed to optimize therapeutic strategies and identify predictive biomarkers for patient selection.

#### 3.1.9 Tryptophan catabolism pathway in RCC

The tryptophan catabolism pathway plays a pivotal role in modulating immune responses within TME. Indoleamine 2,3-dioxygenase 1 (IDO1) is a key catabolic enzyme that degrades tryptophan, a critical amino acid for T-cell proliferation. The depletion of tryptophan in the TME promotes immunosuppression by activating regulatory T cells and MDSCs, fostering an immune-tolerant environment that supports tumor progression (238).

Inhibiting IDO1 has emerged as a promising therapeutic strategy to restore anti-tumor immunity. Epacadostat, a potent and selective IDO1 inhibitor, reduces tryptophan catabolism, thereby enhancing immune surveillance (239). By increasing the proliferation of effector T cells and NK cells while suppressing the expansion of Tregs, epacadostat reactivates anti-tumor immune responses. The combination of epacadostat with pembrolizumab, an anti-PD-1 immune checkpoint inhibitor, has been investigated in a phase I trial involving patients with advanced solid tumors, including RCC. Among 33 patients in the advanced RCC cohort, the ORR was 47% for those with zero-to-one prior treatments and 37% for patients who had undergone two or more prior therapies. The most common treatment-related AE included fatigue, rash, arthralgia, and diarrhea (240).

Building on these promising early-phase results, the combination of epacadostat and pembrolizumab is currently being evaluated as a first-line therapy in a phase III trial (NCT03260894) against the previous standard of care (sunitinib or pazopanib). This ongoing trial aims to establish whether dual IDO1 and PD-1 blockade can provide superior clinical outcomes in advanced RCC patients (241).

Additionally, the RENAVIV randomized phase III trial (NCT03592472) is underway, comparing pazopanib plus abexinostat versus pazopanib plus placebo in first- or second-line settings for patients with advanced RCC. Abexinostat, a histone deacetylase inhibitor, is believed to exert synergistic effects by modulating immune responses and enhancing the efficacy of VEGF inhibitors such as pazopanib. The outcomes of this trial are expected to shed light on the potential benefits of combining metabolic and epigenetic modulation in RCC treatment.

While targeting the tryptophan catabolism pathway offers a novel therapeutic avenue, further studies are required to optimize treatment regimens and identify predictive biomarkers for patient selection. Understanding the complex interactions between metabolic pathways and immune checkpoints is essential for the development of effective combination therapies in RCC.

#### 3.1.10 Exosome-based biomarkers in RCC

In recent years, advances in medical diagnostic technology have significantly improved early detection rates and clinical outcomes in RCC. Exosomes, small extracellular vesicles with a unique bilayer membrane structure, have emerged as a promising non-invasive source of tumor biomarkers. This bilayer provides protection against external Ribonucleases and proteases, thereby preserving the integrity of enclosed messenger RNAs, microRNAs, and functional proteins, and enhancing the stability and sensitivity of these markers for disease diagnosis. Tumor-derived exosomes, particularly their miRNA cargo, have shown potential as biomarkers in the serum and urine of RCC patients, offering valuable targets for early detection, disease monitoring, and therapeutic stratification (242).

## 4 Integration of radiotherapy, chemotherapy, and immunotherapy/targeted therapy in RCC

RCC has traditionally been considered resistant to radiotherapy and chemotherapy. However, recent advances in immunotherapy and targeted therapy have led to renewed interest in combining these modalities to enhance treatment efficacy (243). And we summarized the pivotal studies of combination therapies with immunotherapies and targeted Therapies for the treatment of RCC in **Table 5**. Radiotherapy, while not conventionally used as a primary treatment for RCC, has been shown to induce immunogenic cell death, releasing tumor-associated antigens that promote dendritic cell activation and enhance T-cell priming. Additionally, radiotherapy can modulate the TME by increasing the expression of immune checkpoint molecules such as PD-L1, thereby sensitizing tumors to ICIs (244–246). Clinical studies, including the NIVES trial, have demonstrated that stereotactic body radiotherapy combined with nivolumab results in increased immune activation and durable responses in selected patients (247). Similarly, the RADVAX-RCC trial investigated stereotactic body radiotherapy in combination with dual checkpoint blockade using nivolumab and ipilimumab, showing enhanced T-cell activation and improved response rates. These findings suggest that radiotherapy may act as an immunomodulatory agent that enhances the efficacy of ICIs in RCC (248).

**Table 5:** Selected pivotal studies of combination therapies with Immunotherapies and Targeted Therapies for the treatment of RCC.

Target protein	Experimental arm	Clinical trial	Phase	Response rate	Status
VEGFA and interferon receptor	Bevacizumab + IFN-α	NCT00738530	3	OS 23.3months	Completed
VEGFR, FGFR and mTORC1	Lenvatinib + everolimus	NCT01136733	1\2	PFS 14.6months, OS 25.5months	Completed
PD-1 and VEGFR, PDGFR, c-Kit	Pembrolizumab + axitinib	NCT02853331	3	PFS 15.1months, ORR 59.3%	Active, not recruiting
PD-L1 and VEGFR, PDGFR, c-Kit	Avelumab + axitinib	NCT02684006	3	PFS 13.8months, OS 43.2month	Completed
PD-L1 and VEGF	Atezolizumab + bevacizumab	NCT02420821	3	PFS 11.2months, OS 36.1months	Completed
IL-2 and PD-1	Bempegaldesleukin (NKTR-214) + nivolumab	NCT03729245	3	OS 29month	Terminated
VEGFR, c-Met, AXL and PD-1	Cabozantinib + nivolumab	NCT03141177	3	PFS 16.9months, ORR 55.7%	Active, not recruiting
VEGFR, c-Met, AXL and PD-1 and CTLA-4	Cabozantinib + nivolumab + ipilimumab	NCT03937219	3	No study result	Active, not recruiting
VEGFR, c-Met, AXL and PD-1	Cabozantinib + nivolumab	NCT03793166	3	No study result	Active, not recruiting
VEGFR, FGFR and mTORC1	Lenvatinib + everolimus	NCT02811861	3	PFS14.7months	Active, not recruiting
VEGFR, FGFR and PD-1	Lenvatinib + pembrolizumab	NCT02811861	3	PFS 23.9months	Active, not recruiting
TIGIT and PD-1 and LAG-3 and VEGFR-1	Tiragolumab + Tobolimab + Pembrolizumab + Axitinib	NCT05805501	2	No study results	Active, not recruiting

Beyond its synergy with immunotherapy, radiotherapy has also been investigated in combination with targeted therapy, particularly VEGF-targeted TKIs (249–251). Preclinical and clinical studies have demonstrated that radiotherapy can disrupt tumor vasculature, leading to increased sensitivity to anti-angiogenic agents. For example, stereotactic body radiotherapy combined with sunitinib has been shown to improve local tumor control compared to sunitinib alone, while a combination of stereotactic body radiotherapy and axitinib demonstrated prolonged progression-free survival in advanced RCC patients (252). The ability of VEGF inhibitors to reduce tumor-induced immunosuppression further suggests that their combination with radiotherapy may provide dual benefits of angiogenesis inhibition and enhanced immune priming (253).

Although RCC has historically exhibited low sensitivity to conventional cytotoxic chemotherapy, emerging evidence suggests that specific subtypes, such as nccRCC and sarcomatoid RCC, may benefit from chemotherapy in combination with immunotherapy or targeted therapy (254). Chemotherapy-induced immunogenic effects, including increased antigen presentation and modulation of T-cell responses, may enhance the efficacy of ICIs (254). For example, albumin-bound paclitaxel (nab-paclitaxel) combined with PD-1 inhibitors has shown promising responses in sarcomatoid RCC (255). Additionally, gemcitabine combined with VEGF-TKIs has demonstrated potential in high-risk RCC patients by simultaneously reducing tumor burden and enhancing the anti-angiogenic effects of VEGFR inhibition (256). These findings indicate that while chemotherapy remains a secondary option in RCC, its integration into combination treatment strategies may provide additional therapeutic benefits.

Despite these promising advancements, challenges remain in optimizing the integration of radiotherapy and chemotherapy with immunotherapy and targeted therapy. One of the key challenges is determining the optimal sequencing and dosing of these treatments to maximize efficacy while minimizing toxicity. For instance, excessive immune activation following radiotherapy could lead to increased immune-related AE when combined with ICIs (257, 258). Additionally, the identification of biomarkers predictive of response to combination therapy remains a critical area of investigation. Advances in molecular profiling may help stratify patients who are most likely to benefit from these treatment combinations (259). Future research is also focusing on the incorporation of novel radiosensitizers, DNA damage repair inhibitors such as Poly(ADP-ribose) polymerase inhibitors, and other immunomodulatory agents to further enhance therapeutic outcomes.

In conclusion, the integration of radiotherapy and chemotherapy with immunotherapy and targeted therapy represents a promising avenue for RCC treatment. While additional clinical trials are needed to refine these strategies, current evidence suggests that multi-modal approaches can improve survival outcomes and overcome resistance mechanisms in RCC. The continued exploration of synergistic combinations and biomarker-driven patient selection will be critical in optimizing these treatment paradigms.

## 5 Discussion

The landscape of RCC treatment has evolved considerably, with immunotherapy and targeted therapy emerging as the two most effective approaches for advanced disease (260). While immunotherapy have demonstrated durable responses in a subset of patients, primary and acquired resistance mechanisms, such as immune exclusion, upregulation of alternative immune checkpoints, and metabolic adaptations, continue to limit their efficacy. Similarly, targeted therapies, particularly VEGF and mTOR inhibitors, disrupt tumor angiogenesis and key oncogenic pathways but often lead to adaptive resistance, necessitating combination strategies (261).

Combination approaches have shown promise in overcoming these limitations, particularly the use of ICIs with VEGF-targeted TKIs. This dual strategy aims to normalize tumor vasculature, enhance immune infiltration, and reduce immunosuppressive factors within the tumor microenvironment. Clinical trials have validated the efficacy of combinations such as pembrolizumab plus axitinib and nivolumab plus cabozantinib, demonstrating improved PFS and OS compared to monotherapies. However, these regimens are associated with increased toxicity, raising the need for better patient stratification and biomarker-driven treatment selection (262, 263).

Beyond ICIs and TKIs, adoptive cell therapies, including CAR-T and CAR-NK cells, are being explored in RCC. Despite promising preclinical evidence, challenges such as the immunosuppressive tumor microenvironment, antigen heterogeneity, and limited CAR persistence remain significant hurdles. Advances in gene-modified cell therapies and the development of novel tumor antigens may enhance the feasibility of these approaches. Additionally, other combination modalities, including radiotherapy, chemotherapy, and metabolic inhibitors, hold potential for reshaping the tumor microenvironment to enhance immune response. Radiotherapy, for instance, has been shown to induce immunogenic cell death, which may synergize with ICIs to improve therapeutic efficacy (264).

Moving forward, the integration of multi-omic approaches, including genomics, transcriptomics, and single-cell sequencing, is critical for identifying predictive biomarkers and understanding resistance mechanisms. Precision medicine strategies will play a pivotal role in refining patient selection for specific combination therapies, optimizing treatment sequencing, and minimizing toxicity. Finally, we summarized the FDA-Approved pivotal Immunotherapies and Targeted Therapies for RCC in **Table 6**.

**Table 6:** FDA-Approved pivotal Immunotherapies and Targeted Therapies for RCC.

Targeted therapy	Approval year	First-line/Second-line	Comparator	Target
Aldesleukin (IL-2)	1992	First-Line	None	IL-2 receptor
Sunitinib	2006	First-Line	Interferon	PDGFRα/β, VEGFR1/2/3, KIT, FLT3, RET
Temsirolimus	2007	First-Line	Interferon	mTOR
Pazopanib	2009	First-Line	Placebo	VEGFR, PDGFR, KIT
Pembrolizumab + Axitinib	2019	First-Line	Sunitinib	PD-1, VEGFR
Avelumab + Axitinib	2019	First-Line	Sunitinib	PD-L1, VEGFR
Nivolumab + Ipilimumab	2018	First-Line	Sunitinib	PD-1, CTLA-4
Lenvatinib + Pembrolizumab	2021	First-Line	Sunitinib	VEGFR1-3, FGFR1-4, RET, KIT, PD-1
Nivolumab + Cabozantinib	2021	First-Line	Sunitinib	PD-1, VEGFR, MET, AXL
Sorafenib	2005	Second-Line	Placebo	KIT, PDGFR, RAF, VEGFR
Everolimus	2009	Second-Line	Placebo	mTOR
Axitinib	2012	Second-Line	Sorafenib	KIT, PDGFRβ, VEGFR1/2/3
Nivolumab	2015	Second-Line	Everolimus	PD-1
Cabozantinib	2016	Second-Line	Everolimus	FLT3, MET, RET, VEGFR2
Lenvatinib + Everolimus	2016	Second-Line	Everolimus	VEGFR2
Belzutifan	2023	Second-Line	Everolimus	HIF-2α

## 6 Conclusion

Immunotherapy and targeted therapy have significantly improved outcomes for RCC patients, yet resistance and toxicity remain key challenges. Emerging strategies, such as adoptive cell therapies and metabolic inhibitors, may further enhance treatment efficacy, but their clinical translation requires overcoming substantial biological and technical barriers. Future efforts should focus on identifying robust biomarkers, developing rational combination regimens, and optimizing treatment sequencing to maximize patient benefit. With ongoing advancements in immuno-oncology and precision medicine, a more personalized and durable approach to RCC treatment is on the horizon.

## Glossary

RCC: Renal cell carcinoma
ccRCC: clear-cell renal cell carcinoma
TKI/TKIs: tyrosine kinase inhibitor/tyrosine kinase inhibitors
mTOR: mammalian target of rapamycin
CUP: cancers of unknown primary
PD-1: Programmed Cell Death Protein-1
PD-L1: Programmed Death-Ligand 1
CTLA-4: Cytotoxic T-lymphocyte-associated protein 4
VEGF/VEGFR: Vascular Endothelial Growth Factor/Vascular Endothelial Growth Factor Receptor
nccRCC/nccRCCs: non-clear cell renal cell carcinoma/non-clear cell renal cell carcinomas
ICIs: immune checkpoint inhibitors
DC/DCs: dendritic cell/dendritic cells
IL-2: Interleukin-2
IFN-α: Interferon-alpha
NK cells: natural killer cells
CD: Cluster of Differentiation 279 (或)
PDCD1: Programmed Cell Death 1 (gene)
TCR: T-cell receptor
FDA: Food and Drug Administration
PP2A: Serine/Threonine Protein Phosphatase 2A
PI3K: Phosphatidylinositol 3-kinase
Akt: Protein kinase B
IgG1κ: Immunoglobulin G1 kappa
AE: Adverse Events
LAG-3: Lymphocyte-Activation Gene 3
Ig: immunoglobulin
MHC-II: major histocompatibility complex II
TIM-3: T cell Immunoglobulin and Mucin domain 3
HAVCR2: Hepatitis A Virus Cellular Receptor 2
TIGIT: T cell Immunoreceptor with Immunoglobulin and ITIM domains
PI3K: Phosphatidylinositol 3-kinase
MAPK: Mitogen-Activated Protein Kinase
CIK cells: Cytokine-Induced Killer cells
IFN-γ: Interferon-gamma
MHC: major histocompatibility complex
TCR: T cell receptor
IFNα: Interferon-alpha
ORR: objective response rate
PFS: progression-free survival
OS: overall survival
mRCC: metastatic RCC
MDSCs: myeloid-derived suppressor cells
TME: tumor microenvironment
TILs: Tumor-infiltrating lymphocytes
CR: complete response
REP: Rapid Expansion Protocol
CAR-Ts: Chimeric Antigen Receptor T-Cells
CAR-NKs: Chimeric Antigen Receptor NK Cells
CAR: chimeric antigen receptor
CAIX: Carboxy-anhydrase-IX
AXL: AXL receptor tyrosine kinase
ROR2: Receptor tyrosine kinase-like orphan receptor 2
DNAJB8: DnaJ heat shock protein family (Hsp40) member B8
MUC1: Mucin 1, cell surface associated
c-Met: C-Mesenchymal-Epithelial Transition Factor
EGF/EGFR: Epidermal Growth Factor/Epidermal Growth Factor Receptor
HLA: Human Leukocyte Antigen
VHL: von Hippel-Lindau
HIF2α: hypoxia-inducible factor 2 alpha
PDGF/PDGFR: platelet-derived growth factor/platelet-derived growth factor receptors
pVHL: VHL protein
HIFs: hypoxia-inducible factors
HIF1α: hypoxia-inducible factor 1 alpha
HIF2α: hypoxia-inducible factor 2 alpha
HIF3α: hypoxia-inducible factor 3 alpha
RNAi: RNA interference
FGFR: Fibroblast Growth Factor Receptor
TGFβ: Transforming Growth Factor-beta
ALK1: Activin Receptor-Like Kinase 1
aRCC: advanced RCC
MEK: ERK Kinase
ERK: Extracellular Signal-Regulated Kinase
JAK: Janus Kinase
STAT: Signal Transducer and Activator of Transcription
T: telaglenastat
E: everolimus
P: placebo
C: cabozantinib
ATP: Adenosine Triphosphate
IDO1: Indoleamine 2,3-dioxygenase 1.
