# Supplementary material for: Advances in immunotherapy and targeted therapy for advanced clear-cell renal cell carcinoma: current strategies and future directions
Source: Front Immunol. 2025 Jul 3;16:1582887. doi: 10.3389/fimmu.2025.1582887 (PMC12267214; doi:10.3389/fimmu.2025.1582887)
Supplement: Supplementary file 1 [file DataSheet1.pdf]

Supplementary 1

Materials and Methods

Literature Search Strategy:

A systematic literature search was conducted using the following databases: PubMed, Web of Science and ClinicalTrials.gov, covering publications from January 2000 to 2025. The search keywords included combinations of:

"clear-cell renal cell carcinoma,"

"ccRCC,"

"immunotherapy,"

"targeted therapy,"

"immune checkpoint inhibitors,"

"VEGF inhibitors,"

"mTOR inhibitors,"

"clinical trials,"

"advanced RCC,"

"adjuvant therapy."

Inclusion Criteria:

1. Studies focusing on advanced ccRCC (Phase II/III clinical trials, observational studies, meta-analyses, and seminal preclinical studies).
2. Articles published in English.
3. Research evaluating FDA-approved therapies or investigational agents with robust clinical evidence.
4. Studies reporting overall survival (OS), progression-free survival (PFS), or objective response rates (ORR).

Exclusion Criteria:

1. Case reports, editorials, or non-peer-reviewed articles.
2. Articles lacking primary outcome data or mechanistic insights.
3. Preclinical studies without translational relevance to human ccRCC.

Supplementary Table 1: Limitations of Current Immunotherapy and Targeted Therapy Regimens in Advanced ccRCC

Category	Regimen	Key Limitations
**Immunotherapy**		
	IL-2/IFN-α	Severe toxicity (e.g., vascular leak syndrome)
	PD-1/PD-L1 inhibitors	Limited efficacy in CNS metastases
	CTLA-4 inhibitors	High immune-related adverse events (e.g., colitis, hepatitis)
	LAG-3/TIM-3/TIGIT inhibitors	Limited clinical data; unclear biomarkers for patient selection
	CAR-T/CAR-NK cells	Antigen heterogeneity; immunosuppressive TME; limited persistence in vivo
	DC vaccines	Low response rates (10-20%); tumor-mediated immune suppression
**Targeted Therapy**		
	HIF2α inhibitors (Belzutifan)	Anemia; limited efficacy in non-VHL-mutated tumors
	VEGF-TKIs (Sunitinib, Cabozantinib)	Adaptive resistance; hypertension, fatigue, hand-foot syndrome
	mTOR inhibitors (Everolimus)	Hyperglycemia, stomatitis; paradoxical HIF2α stabilization via feedback loops
	Glutaminase inhibitors (Telaglenastat)	Modest PFS benefit; high toxicity in combination regimens
	AXL inhibitors	Lack of validated predictive biomarkers; limited single-agent

	(Baitraxcept)	activity
	Adenosine receptor antagonists	Variable efficacy; complex interplay with TME metabolism
	IDO1 inhibitors (Epacadostat)	Failed Phase III trials (no OS benefit); compensatory tryptophan catabolism
